# Characteristics of medical costs and resource use in patients with rheumatoid arthritis treated with and without glucocorticoids

**DOI:** 10.1371/journal.pone.0329313

**Published:** 2025-07-30

**Authors:** Eiichi Tanaka, Eisuke Inoue, Ryoko Sakai, Katsuhiko Iwasaki, Ayako Shoji, Masayoshi Harigai

**Affiliations:** 1 Department of Rheumatology, Tokyo Women’s Medical University School of Medicine, Shinjuku, Tokyo, Japan; 2 Showa University Research Administration Center (SURAC), Showa University, Shinagawa, Tokyo, Japan; 3 Department of Public Health and Epidemiology, Meiji Pharmaceutical University, Kiyose-city, Tokyo, Japan; 4 Division of Science and Analytics, Medilead Inc., Shinjuku, Tokyo, Japan; 5 Healthcare Consulting, Inc., Chiyoda, Tokyo, Japan; 6 Department of Health Economics and Outcomes Research, Graduate School of Pharmaceutical Sciences, The University of Tokyo, Bunkyo, Tokyo, Japan; Nippon Medical School, JAPAN

## Abstract

**Objectives:**

To evaluate medical costs and resource use in patients with rheumatoid arthritis (RA) treated with and without oral or injectable glucocorticoids (GCs) as part of their initial treatment with disease-modifying antirheumatic drugs (DMARDs).

**Methods:**

Patients included in the Japan Medical Data Center health insurance claims database and diagnosed with RA were considered. The date of the first prescription of a DMARD (index date) after an observable 6-month period (baseline) was used to define follow-up (12 months post-index date) periods. Patients with at least one GC prescription in the follow-up period were included in the GC group, and patients without a GC prescription in the follow-up period were classified as the non-GC group. The primary endpoints were costs for drugs, treatments, and materials per patient in the follow-up period. Drugs were divided into medications for RA or for adverse events (AEs). The secondary endpoints were proportions of patients using the subcategories of each resource. The incidence of hospitalization during the follow-up period was evaluated.

**Results:**

A total of 1,670 and 1,487 patients with median ages of 51.0 and 50.0 years were evaluated in the GC and non-GC groups, respectively. The costs for drugs, treatments, and materials were significantly higher in the GC group compared with the non-GC group (GC/ non-GC; drug costs for RA and AEs, 2,818 USD/ 1,882 USD; drug costs for RA only, 2,697 USD/ 1,805 USD; treatment costs, 2,365 USD/ 1,860 USD; material costs, 112 USD/ 77 USD; *P* < 0.05). The resource use in almost all drug and treatment subcategories was higher in the GC group. The incidence of hospitalization was also higher in the GC group.

**Conclusions:**

Patients with RA treated with GCs in the first year after starting DMARDs tended to use more resources and have higher medical costs than patients not treated with GCs.

## Introduction

Rheumatoid arthritis (RA) is a chronic, progressive autoimmune disease. The primary goal of treatment is to achieve remission and maintain it. Conventional synthetic disease-modifying antirheumatic drugs (csDMARDs) are recommended as the first-line treatment in all patients after diagnosis. The 2022 update of the European League Against Rheumatism (EULAR) recommendations for the management of RA also recommends co-administration of glucocorticoids (GCs) as bridging therapy in patients with RA when initiating or changing csDMARDs because these drugs show fast-acting effects that can improve the success rate of disease-modifying antirheumatic drugs (DMARDs) and help to avoid disease flare-ups [1]. However, because GCs are known to be associated with serious adverse drug reactions, such as an increased risk of infection, diabetes, hypertension, weight gain, and osteoporosis, the EULAR recommendations state that GC dosage should be tapered and GC administration should be discontinued as rapidly as is clinically feasible [1]. In contrast, the 2021 update of the American College of Rheumatology (ACR) Guideline for the Treatment of Rheumatoid Arthritis does not recommend GCs, even in the short term, and allows them to be temporally used only in the shortest duration with minimum doses [2]. The 2020 update of the Japan College of Rheumatology Clinical Practice Guidelines for the Management of RA is similar to that of the ACR of Rheumatology guidelines. The guidelines recommend GCs as adjunctive drugs and reported that it is preferable to use them for a short time with minimum doses for pain relief, even if they are temporally used [3].

Many studies have described an inappropriate use of GCs that was likely to be a safety concern. For example, some studies found that GCs were often used before DMARDs were initiated or without any additional DMARDs therapy [4,5]. Another study found that a higher proportion of patients with RA used GCs than would have been expected on the basis of the proportion of patients with high disease activity [6]. Moreover, reports based on a large cohort study revealed that an inappropriate long-term use of GCs was associated with functional disabilities in patients with RA [7,8]. On the other hand, information on best practices for prescribing GCs, such as appropriate dosages and durations, still requires further research [9], and concerns about adverse events (AEs) are likely to limit their use even in appropriate situations. The most recent version of the EULAR recommendations and the Japanese guidelines include a statement about the use of GCs in combination with csDMARDs. Consequently, Japanese patients with RA may not necessarily be prescribed GCs even when they have an indication for the drug. Poor disease management after the initial diagnosis of RA can affect the overall use of health services and may subsequently lead to an increase in the economic burden on patients. However, there are few studies focused on the relationship between medical costs and GC use in the early stage after onset of RA.

Therefore, this study aimed to compare medical costs and resource use in patients with RA who did or did not receive GCs as part of their initial csDMARDs therapy.

## Materials and methods

### Study design

This was a retrospective cohort study that compared medical costs and resource use between patients with RA who were prescribed GCs and those who were not treated with GCs. The protocol including this study was approved by the Ethics Committee of Tokyo Women’s Medical University on November 21, 2018 (Ethical Approval Number: 4991). Informed consent was not applicable for this study, based on the ethical guidelines for medical and health research involving human participants issued by the Ministry of Health, Labor and Welfare and Ministry of Education, Culture, Sports, Science and Technology of Japan (revised on February 28, 2017). This study started on March 9, 2020 and ended on February 26, 2024.

### Data source

Data for the study were derived from a large-scale health insurance claims administrative database maintained by the Japan Medical Data Center (JMDC Inc.) [10]. The JMDC has collected anonymized patient data since 2005 from health care insurance claims made by individuals working at medium- to large-scale Japanese companies and their dependents; data include patient characteristics, such as age and sex, outpatient, inpatient, and prescription charges, and International Classification of Diseases 10th revision (ICD-10) diagnoses. The database summarizes all claims information for each patient, regardless of where they receive treatment, enabling researchers to analyze long-term follow-up data, as long as there is no change in the insurance system to which patients belong. As of December 2018, the database comprised data from 5.6 million Japanese individuals [10] Compared with other claims database, the JMDC database allows to follow treatment histories after changing hospitals but partially covers individuals aged 65 and over [10,11].

### Study population

Patients whose data were extracted from the JMDC database were eligible for inclusion in this study provided they met all of the following inclusion criteria: 1) having a confirmed diagnosis of RA on the basis of the ICD-10 code assigned to each patient (see S1 Table); 2) the date of their first prescription of a DMARD (index date) falling in the time period between January 1, 2012 to December 31, 2017 (see S2 Table); 3) having data available for the baseline period (6 months before the index date) and the follow-up period (12 months post-index date).

The DMARDs included in the eligibility criteria were conventional synthetic DMARDs (csDMARDs), including methotrexate (MTX), biological DMARDs (bDMARDs), and targeted synthetic DMARDs (tsDMARDs). The exclusion criteria for this study were as follows: having a confirmed diagnosis of any of the diseases shown in S1 Table in the baseline or follow-up periods; having any prescription of a DMARD in the baseline period; having any prescription of a GC before the first diagnosis of RA in the baseline period.

**Fig 1:**
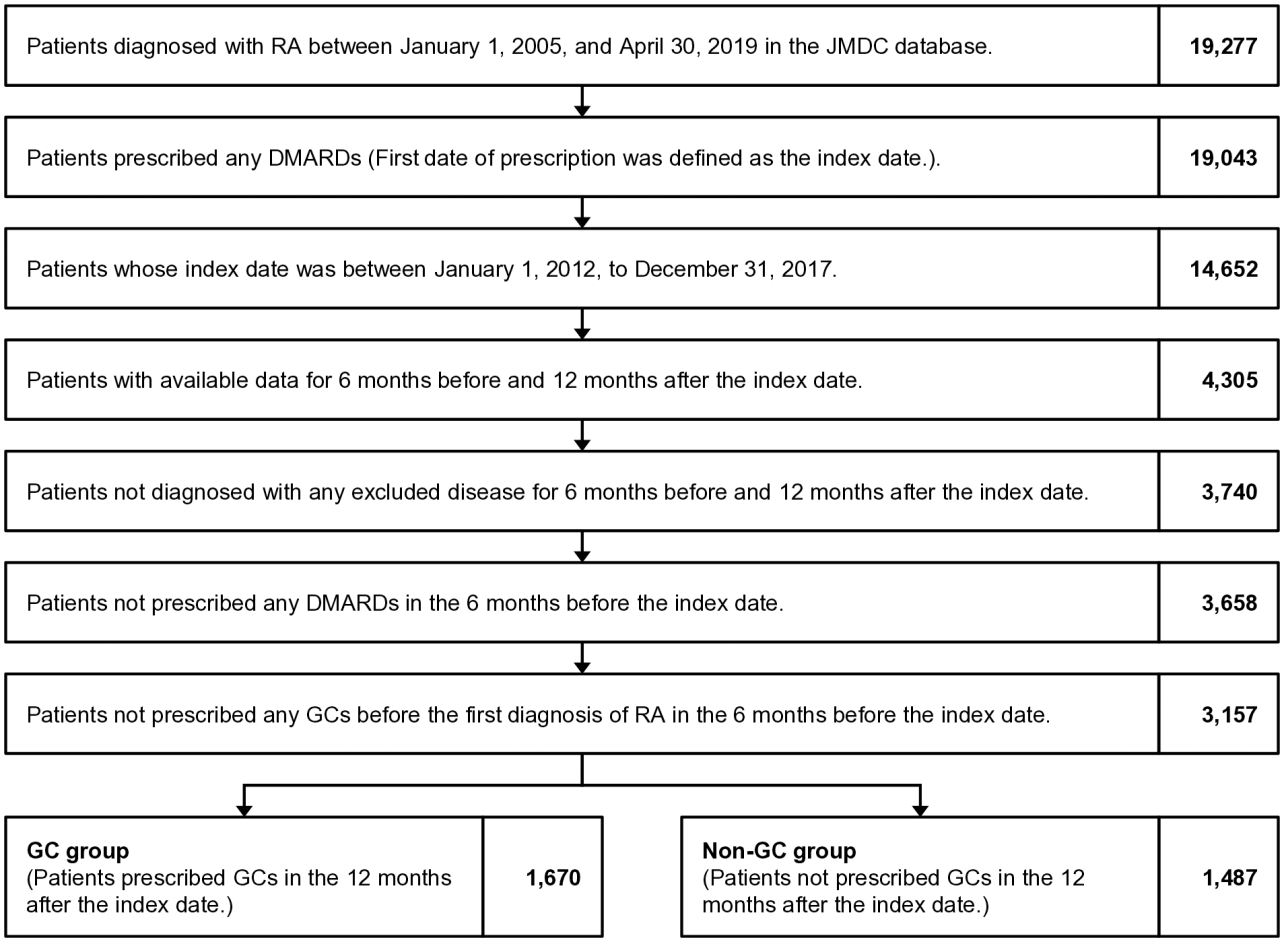
Flowchart of patient extraction. GC, glucocorticoid; DMARD, disease-modifying antirheumatic drug; JMDC, Japan Medical Data Center; RA, rheumatoid arthritis.

Patients with at least one prescription of any oral or injectable GC (see S2 Table) in the follow-up period were defined as the GC group, and patients with no prescription of any GC in the follow-up period were defined as the non-GC group (see Fig 1).

### Endpoints

The primary endpoint was defined as the total medical costs including those for drugs, treatments, and materials in the follow-up period. Medical costs in Japanese Yen (JPY) were converted into US Dollars (USD) where 1 USD equaled 105 JPY in 2020. Drugs included those related to RA (namely, DMARDs, GCs, and analgesics; see S2 Table) and those prescribed for symptoms related to GC use. We included the costs of drugs for GC use-related AEs (GC-related AEs, hereafter) (antibiotics and anti-osteoporotic drugs were considered; S3 Table) because they may lead to higher medical costs. We performed two different analyses of drug costs, namely, with and without drugs for GC-related AEs. Treatment and material costs included costs for all medical care in the follow-up period (see S4 and S5 Tables), although some costs were excluded, such as those for cataract operations and childbirth-related care.

Secondary endpoints included medical costs and the use of drugs, treatments, and materials in the follow-up period. The medical care analyzed for the secondary endpoints was similar to that analyzed for the primary endpoints. To evaluate drug use, we assessed the number and proportion of patients who used each eligible drug and the maximum dose of MTX per week in the follow-up period. To evaluate treatment resource use in the follow-up period, we assessed the number and proportion of patients who used each resource and the number of days on which they used it.

Additional endpoints were the incidence of clinical events in the follow-up period. To evaluate the incidence of clinical events, we used the Kaplan-Meier method to compare the relationship between the time from the index date to event occurrence and the rate of event occurrence between the groups. We focused on three clinical events: the time from the index date to event occurrence, the rate of event occurrence, and all-cause hospitalization. We defined the date of the first event after the index date as the date of event occurrence. Patients who had already experienced the event during the baseline period, including at the index date, were excluded from the analysis.

### Statistical analysis

For medical costs, days of resource use, maximum MTX dose (mg/per week), age, Charlson comorbidity index (CCI) [12], and disease duration, we calculated means (± standard deviation, SD) or medians (interquartile range, IQR) and performed the Wilcoxon rank sum test to assess differences in each variable between the GC and non-GC groups. For drug and treatment resource use, we calculated the number and proportion of patients and performed the chi-squared test to assess group differences. For the incidence of hospitalization, the Kaplan-Meier method was used to estimate cumulative incidence, and the log-rank test was used to assess group differences. Results were considered statistically significant if the *p*-value was less than 0.05. All comparisons used two-sided tests, and all statistical analyses were performed with Statistical Analysis Software R (version 3.6.0).

## Results

### Patient characteristics

Of the 5.6 million individuals, 19,227 were diagnosed with RA in the JMDC database. We identified 1,670 eligible patients in the GC group and 1,487 in the non-GC group (Fig 1). Based on the index date, we found differences between groups regarding the CCI, disease duration, and treatment with MTX and other csDMARDs (Table 1).

**Table 1:** Patient characteristics.

Patient characteristic		Group		
		GC (N = 1,670)	Non-GC (N = 1,487)	*P*-value
Sex, n (%)	Female	1,191 (71.3)	1,091 (73.4)	0.213
csDMARDs at index date, n (%)	Total	1,621 (97.1)	1,441 (96.9)	0.875
	MTX	1,140 (68.3)	1,103 (74.2)	< 0.001
	Others	547 (32.8)	393 (26.4)	< 0.001
bDMARDs at index date, n (%)	Total	79 (4.7)	77 (5.2)	0.619
	TNFi	57 (3.4)	52 (3.5)	0.975
	IL6i	12 (0.7)	18 (1.2)	0.216
	T-cell	10 (0.6)	7 (0.5)	0.805
tsDMARDs at index date, n (%)		0 (0.0)	0 (0.0)	NA
Age, median (IQR), years old		51.0 (43.0-57.0)	50.0 (43.0-57.0)	0.594
CCI, median (IQR)		2.0 (1.0-4.0)	2.0 (1.0-4.0)	0.032
Disease duration*, median (IQR), month		1.3 (0.7-11.0)	1.2 (0.6-9.8)	0.007
Number of patients, n**		1,140	1,103	NA
Dose of MTX at index date, mean (SD), mg/week		6.2 (2.0)	6.3 (1.9)	0.402

* Time from the diagnosis of RA to the first prescription of DMARDs.

** The number of patients who were prescribed MTX at index date, namely, the denominator for calculating the mean dose of MTX.

bDMARD, biological disease-modifying antirheumatic drug; CCI, Charlson comorbidity index; GC, glucocorticoid; csDMARD, conventional synthetic disease-modifying antirheumatic drug; IL6i, interleukin-6 inhibitor; IQR, interquartile range; MTX, methotrexate; NA, not applicable; RA, rheumatoid arthritis; SD, standard deviation; T-cell, selective T-cell co-stimulation modulator; TNFi, tumor necrosis factor α inhibitor; tsDMARD, targeted synthetic disease-modifying antirheumatic drug.

*P*-value: Chi-square test for sex, csDMARDs at index date, bDMARDs at index date, and tsDMARDs at index date; Wilcoxon rank sum test for age, CCI, disease duration, and dose of MTX at index date.

### Medical costs

The total medical costs and their categories (namely, drugs, treatments, and materials) are shown in Table 2. Regardless of whether costs related to AEs were included or not, the total medical costs were significantly higher in the GC rather than the non-GC group (Table 2). The drug costs with and without those for GC-related AE, treatment costs, and material costs were also significantly higher in the GC rather than the non-GC group (Table 2).

**Table 2:** Annual medical costs per patient.

Type of medical cost		Medical costs per patient, mean (SD), USD/year		
		GC (N = 1,670)	Non-GC (N = 1,487)	*P*-value
Total medical costs	Total	5,295 (6,797)	3,817 (5,324)	< 0.001
	For RA only*	5,174 (6,718)	3,740 (5,213)	< 0.001
Drug costs	Total	2,818 (4,645)	1,882 (3,780)	< 0.001
	For RA only*	2,697 (4,611)	1,805 (3,655)	< 0.001
Treatment costs	Total	2,365 (3,455)	1,860 (2,899)	< 0.001
Material costs	Total	112 (1,102)	77 (722)	0.001

GC, glucocorticoid; DMARD, disease-modifying antirheumatic drug; RA, rheumatoid arthritis; SD, standard deviation.

1 USD = 105 JPY in 2020.

*P*-value: Wilcoxon rank sum test.

* Only includes costs for DMARDs, GCs, and analgesics; costs for antibiotics and anti-osteoporotic drugs were excluded.

The drug costs for each type of drug are shown in Table 3. bDMARDs made up the highest proportion of total drug costs in both groups, and costs for tumor necrosis factor inhibitors (TNFi) were also particularly high (Table 3). The cost of DMARDs except MTX and tsDMARDs and costs of analgesics except opioids were significantly higher in the GC rather than the non-GC group. Costs of drugs for infection including antibacterial drugs, were also significantly higher in the GC than the non-GC group; however, the costs of antifungal drugs were lower in the GC group (Table 3).

**Table 3:** Annual drug costs per patient.

Type of drug		Drug costs per patient, mean (SD), USD/year		
		GC (N = 1,670)	Non-GC (N = 1,487)	*P*-value
csDMARDs	Total	615 (856)	489 (576)	< 0.001
	MTX	321 (241)	320 (247)	0.714
	Others	294 (858)	169 (529)	< 0.001
bDMARDs	Total	1,824 (4,464)	1,165 (3,573)	< 0.001
	TNFi	1,290 (3,912)	840 (3,146)	< 0.001
	IL6i	336 (1,609)	211 (1,401)	< 0.001
	T-cell	199 (1,493)	114 (1,162)	0.009
tsDMARDs		15 (338)	3 (117)	0.135
GC		45 (57)	0 (0)	< 0.001
Analgesics	Total	197 (289)	148 (224)	< 0.001
	AAP	2 (10)	1 (5)	0.001
	AAP/Opioids	11 (91)	4 (46)	< 0.001
	NSAIDs	164 (221)	132 (196)	< 0.001
	Opioids	3 (53)	1 (10)	0.372
	Others	17 (101)	10 (76)	< 0.001
Anti-infective drugs	Total	61 (234)	57 (969)	< 0.001
	Antibacterial drugs	35 (120)	19 (64)	< 0.001
	Antifungal drugs	16 (159)	34 (964)	0.014
	Antiviral drugs	10 (107)	4 (63)	0.084
	Antiparasitic drugs	0 (1)	0 (0)	0.298
Anti-osteoporotic drugs		60 (214)	20 (97)	< 0.001

AAP, acetaminophen; bDMARD, biological disease-modifying antirheumatic drug; GC, glucocorticoid; csDMARD, conventional synthetic disease-modifying antirheumatic drug; IL6i, interleukin-6 inhibitor; MTX, methotrexate; NSAID, non-steroidal anti-inflammatory drug; SD, standard deviation; T-cell, selective T-cell co-stimulation modulator; TNFi, tumor necrosis factor α inhibitor; tsDMARD, targeted synthetic disease-modifying antirheumatic drug.

1 USD = 105 JPY in 2020.

*P*-value: Wilcoxon rank sum test.

The treatment costs are shown in Table 4. In both groups, examination costs accounted for the highest proportion of the total treatment costs (Table 4). With some exceptions, the cost of each treatment was significantly higher in the GC than the non-GC group (Table 4).

**Table 4:** Annual treatment costs per patient.

Type of treatment	Treatment costs per patient, mean (SD), USD/year		
	GC (N = 1,670)	Non-GC (N = 1,487)	*P*-value
Outpatient	286 (194)	234 (148)	< 0.001
Hospitalization	191 (1,412)	122 (1,222)	< 0.001
Medical management	124 (186)	96 (163)	< 0.001
Home health care	70 (314)	60 (353)	0.003
Examination	942 (670)	778 (458)	< 0.001
Imaging	208 (314)	152 (224)	< 0.001
Medication	136 (80)	114 (68)	< 0.001
Injection	37 (102)	21 (87)	< 0.001
Rehabilitation	54 (266)	53 (614)	< 0.001
Psychiatric specialty therapy	17 (108)	12 (75)	0.320
Procedure	62 (1,162)	56 (1,165)	< 0.001
Surgery	169 (961)	117 (811)	0.009
Anesthesia	38 (236)	25 (199)	< 0.001
Radiation therapy	8 (316)	1 (40)	0.326
Pathological diagnosis	22 (72)	14 (53)	0.010
Others	0 (1)	0 (1)	0.998

GC, glucocorticoid; SD, standard deviation.

1 USD = 105 JPY in 2020.

*P*-value: Wilcoxon rank sum test.

### Medical resource use

Table 5 shows the number and proportion of patients who used drugs for RA or AEs in the follow-up period. Although the proportions of patients using MTX were similar between the two groups, those using the RA-related drugs, csDMARDs other than MTX, all types of bDMARDs, and analgesics other than opioids, were significantly higher in the GC than the non-GC group (Table 5). The use of antibacterial, antifungal, and anti-osteoporotic drugs was significantly higher in the GC than the non-GC group (Table 5). The maximum dose of MTX in the follow-up period was significantly higher in the GC than the non-GC group (mean [SD]: 6.9 (3.9) mg/week vs. 6.5 (3.7) mg/week (*P* < 0.001)).

**Table 5:** Number and proportion of patients with drug use.

Type of drug		Drug use, n (%)		
		GC (N = 1,670)	Non-GC (N = 1,487)	*P*-value
csDMARDs	Total	1,635 (97.9)	1,447 (97.3)	0.328
	MTX	1,481 (88.7)	1,315 (88.4)	0.870
	Others	790 (47.3)	551 (37.1)	< 0.001
bDMARDs	Total	342 (20.5)	181 (12.2)	< 0.001
	TNFi	252 (15.1)	129 (8.7)	< 0.001
	IL6i	93 (5.6)	40 (2.7)	< 0.001
	T-cell	40 (2.4)	17 (1.1)	0.012
tsDMARDs		5 (0.3)	1 (0.1)	0.278
GC		1,670 (100.0)	0 (0.0)	< 0.001
Analgesics	Total	1,512 (90.5)	1,274 (85.7)	< 0.001
	AAP	379 (22.7)	273 (18.4)	0.003
	AAP/Opioids	84 (5.0)	37 (2.5)	< 0.001
	NSAIDs	1,459 (87.4)	1,214 (81.6)	< 0.001
	Opioids	16 (1.0)	10 (0.7)	0.491
	Others	198 (11.9)	101 (6.8)	< 0.001
Antibiotics	Total	1,086 (65.0)	873 (58.7)	< 0.001
	Antibacterial drugs	1,022 (61.2)	800 (53.8)	< 0.001
	Antifungal drugs	133 (8.0)	86 (5.8)	0.019
	Antiviral drugs	172 (10.3)	129 (8.7)	0.136
	Antiparasitic drugs	5 (0.3)	8 (0.5)	0.443
Anti-osteoporotic drugs		341 (20.4)	95 (6.4)	< 0.001

AAP, acetaminophen; bDMARD, biological disease-modifying antirheumatic drug; GC, glucocorticoid; csDMARD, conventional synthetic disease-modifying antirheumatic drug; IL6i, interleukin-6 inhibitor; MTX, methotrexate; NSAID, non-steroidal anti-inflammatory drug; T cell, selective T-cell co-stimulation modulator; TNFi, tumor necrosis factor inhibitor; tsDMARD, targeted synthetic disease-modifying antirheumatic drug.

*P*-value: Chi-square test.

Table 6 shows the number and proportion of patients who received each type of treatment in the follow-up period. With some exceptions, the proportion of patients who received each treatment was significantly higher in the GC than the non-GC group (Table 6).

**Table 6:** Number and proportion of patients with treatment resource use.

Type of treatment	Treatment resource use, n (%)		
	GC (N = 1,670)	Non-GC (N = 1,487)	*P*-value
Outpatient	1,670 (100.0)	1,483 (99.7)	0.105
Hospitalization	199 (11.9)	93 (6.3)	< 0.001
Medical management	1,365 (81.7)	1,143 (76.9)	0.001
Home health care	213 (12.8)	138 (9.3)	0.002
Examination	1,665 (99.7)	1,473 (99.1)	0.036
Imaging	1,414 (84.7)	1,191 (80.1)	0.001
Medication	1,670 (100.0)	1,483 (99.7)	0.105
Injection	958 (57.4)	416 (28.0)	< 0.001
Rehabilitation	209 (12.5)	120 (8.1)	< 0.001
Psychiatric specialty therapy	82 (4.9)	62 (4.2)	0.363
Procedure	744 (44.6)	517 (34.8)	< 0.001
Surgery	170 (10.2)	112 (7.5)	0.011
Anesthesia	160 (9.6)	74 (5.0)	< 0.001
Radiation therapy	5 (0.3)	2 (0.1)	0.546
Pathological diagnosis	280 (16.8)	205 (13.8)	0.023
Others	37 (2.2)	33 (2.2)	1.000

GC, glucocorticoid.

*P*-value: Chi-square test.

Table 7 shows the number of days on which patients received each type of treatment in the follow-up period. The numbers of days for each type of treatment were significantly higher in the GC than the non-GC group (Table 7).

**Table 7:** Number of days on which patients received various types of treatment.

Type of treatment	Treatment resource use, mean (SD), days/year		
	GC (N = 1,670)	Non-GC (N = 1,487)	*P*-value
Outpatient	25.1 (18.1)	20.2 (13.8)	< 0.001
Hospitalization	1.7 (9.6)	0.9 (6.6)	< 0.001
Imaging	3.3 (5.2)	2.3 (2.9)	< 0.001
Rehabilitation	2.4 (10.9)	1.4 (8.6)	< 0.001
Procedure	3.6 (11.5)	2.3 (8.8)	< 0.001
Surgery	0.1 (0.5)	0.1 (0.4)	0.009

GC, glucocorticoid; SD, standard deviation.

*P*-value: Wilcoxon rank sum test.

### Incidence of hospitalization in the follow-up period

**Fig 2:**
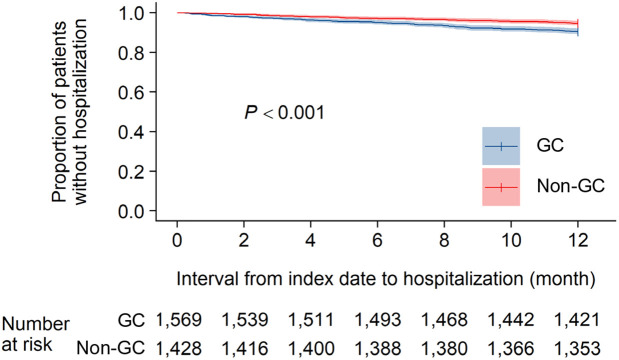
Kaplan-Meier curves of clinical events in the 12-month follow-up period. The areas filled with semi-transparent colors around the curves indicate the 95% confidence intervals. The vertical bars on the curves indicate the censorings. The *p*-value was calculated by a log-rank test. GC, glucocorticoid.

The incidences of hospitalization in the 12-month follow-up period are shown as Kaplan-Meier curves (Fig 2). In the GC group, hospitalization occurred earlier and more frequently compared with the non-GC group.

## Discussion

In this study, we evaluated medical costs in the 12 months after patients were diagnosed with RA and started treatment with DMARDs and compared the costs between patients who did and did not receive concomitant GC treatment. We found that the total medical costs and their categories were higher in the GC than the non-GC group.

Almost all costs in the drug and treatment subcategories were higher in the GC than the non-GC group, with some exceptions. These results suggest that patients in the GC group required more medical care than those in the non-GC group, which is likely associated with the higher disease activity in the GC groups that we could not be observed in the claims database. Recent studies have reported that GC usage and high disease activity in the early stage after RA onset were independent negative predictors of the subsequent achievement of disease remission [13,14]. In the present study, the GC group had higher costs for and more prevalent use of RA-related drugs, such as non-MTX csDMARDs, bDMARDs, and analgesics compared with the non-GC group. Although we were unable to clarify the relationship between disease activity at baseline between the two groups because of the lack of information in this type of database, our present findings suggest that more patients in the GC group had difficulty in controlling RA than those in the non-GC group and then might had started the treatment with GC.

This study analyzed patient data for only 12 months immediately after initiation of treatment with DMARDs. The Kaplan-Meier survival curves in the 12-month follow-up period showed that hospitalization occurred at a constant rate in both groups but earlier and more often in the GC group compared with the non-GC group. These findings indicate that patients requiring GCs as part of their initial therapy are more likely to experience hospitalization throughout the treatment period.

A previous study has shown that chronic exposure to GCs increases the risk of GC-related AEs, leading to an increase in AE-related medical costs [15]. This study estimated that the high use of GCs (cumulative dose > 1800 mg) would lead to additional costs of approximately 3,528 USD for AEs per patient per year (370,000 JPY at a conversion rate of 1 USD = 105 JPY). Consistent with those earlier findings, our present study focused on differences in medical costs in patients with and without exposure to GC. However, we showed clearly that the medical costs for RA treatment itself are also higher in patients treated with GCs, in addition to increased costs other than RA treatment. Regarding these differences in medical costs, we showed that almost all costs and resource use for the subcategories of drugs, treatments, and materials were higher in the GC group compared with the non-GC group. However, we have not directly shown whether each GC was used appropriately or whether it contributed to control disease activity. To assess the value of GC use for initial RA treatment, the cost-effectiveness of GC use should be evaluated in future studies, considering benefits such as improvement in disease activity, physical function, and quality of life.

When considering RA medical costs with and without GC from the perspective of comorbidities, the higher the baseline CCI score, the higher the annual medical costs, which explains the higher the annual medical costs in the GC group than in the non-GC group (see S6 Table). In addition, the GC group had more expenses than the non-GC group for many types of comorbidities, such as cardiovascular disease, chronic kidney disease, diabetes, or lymphoma (see S6 Table).

Furthermore, Generally, patients who require GCs are generally expected to have a greater mental burden if their physical burden is greater, and the number of patients who require mental care is also expected to increase. However, the results of this study showed that the costs of mental care in the GC group did not increase in the GC group. (Table 6). This finding suggests that future research focusing on this issue is necessary, including whether mental disorders requiring treatment may be overlooked.

### Limitations

The present study had some limitations. First, because claims data do not provide information about disease activity and physical function in patients with RA, we could not clarify whether disease severity differed between the GC and non-GC groups, which may be a potential confounding factor. Second, because only corporate employees and their dependents are registered in the JMDC database, the proportion of elderly people aged 65 years and over, who are the non-working population, is relatively small, although the elderly -onset RA has recently been the focus [16]. Furthermore, the median age of our cohort was slightly lower than that of a previous report [17]. Third, although ICD-10 codes and DMARDs usage were employed to identify patients with RA, the validity of this approach has not been confirmed in the JMDC claims database. Fourth, we did not adjust for confounders between groups because disease activity is the most important factor associated with the use of GCs; however, no information on this was contained in the claims database in Japan.

## Conclusions

Patients with RA who were treated with GCs in the first year after starting DMARDs therapy incurred significantly higher medical costs and expended greater medical resources than those without GCs.

## Supporting information

S1 Table ICD-10 codes. (PDF)

S2 Table Items included in drug costs for treatment of RA. (PDF)

S3 Table Items included in drug costs for treatment of adverse events. (PDF)

S4 Table Items included in treatment costs. (PDF)

S5 Table Items included in material costs. (PDF)

S6 Table The number and percentage of eligible patients and annual medical costs per patient by the disease name included in the Charlson comorbidity index and Charlson comorbidity index score. (PDF)
