# Supplementary material for: Characteristics of medical costs and resource use in patients with rheumatoid arthritis treated with and without glucocorticoids
Source: PLoS One. 2025 Jul 30;20(7):e0329313. doi: 10.1371/journal.pone.0329313 (PMC12310026; doi:10.1371/journal.pone.0329313)
Supplement: S1 Table — (PDF) [file pone.0329313.s001.pdf]

S1 Table. ICD-10 codes

Disease	ICD-10 codes
Included as RA	M050, M051, M052, M053, M058, M059, M060, M062, M063, M064, M068, M069
Excluded as exclusion disease	D477, K500, K501, K508, K509, K510, K512, K513, K515, K518, K519, L400, L401, L402, L403, L404, L405, L408, L409, L732, M061, M080, M081, M082, M084, M088, M089, M303, M314, M316,

ICD-10, International Classification of Diseases 10th Revision; RA, rheumatoid arthritis
