# Supplementary material for: Characteristics of medical costs and resource use in patients with rheumatoid arthritis treated with and without glucocorticoids
Source: PLoS One. 2025 Jul 30;20(7):e0329313. doi: 10.1371/journal.pone.0329313 (PMC12310026; doi:10.1371/journal.pone.0329313)
Supplement: S2 Table — (PDF) [file pone.0329313.s002.pdf]

S2 Table. Items included in drug costs for treatment of RA

Drug type			Ingredient name
DMARDs	csDMARDs	MTX	Methotrexate
		nonMTX	Actalit, Aura no fin, Bucillamine, Gold sodium thiomalate, Iguratimod, Leflunomide, Lobenzarit disodium, Mizoribine, Penicillamine, Salazosulfapyridine, Tacrolimus
	bDMARDs	TNFi	Adalimumab, Certolizumab pegol, Etanercept, Golimumab, Infliximab
		IL6i	Salilumab, Tocilizumab
		T-cell	Abatacept
	tsDMARDs		Ballicitinib, Peficitinib hydrobromide, Tofacitinib citrate
GCs			Betamethasone, Cortisone acetate, Dexamethasone, Dexamethasone palmitate, Dexamethasone sodium phosphate, Hydrocortisone, Hydrocortisone sodium succinate, Methylprednisolone, Methylprednisolone acetate, Prednisolone, Prednisolone sodium succinate, Triamcinolone, Triamcinolone acetonide
Analgesics	AAP		Acetaminophen
	AAP/Opioids		Tramadol hydrochloride/acetaminophen
	NSAIDs		Acemethacin, Adrenal gland extract/heparin-like substance combination, Ampiroxicam, aspirin, Aspirin/Dialuminate, Celecoxib, Diclofenac sodium, Esflurbiprofen/mint oil, Etodolac, Felbinac, Flufenamic acid aluminum, Flurbiprofen, Ibuprofen, Indomethacin, Indomethacin farnesyl, Ketoprofen, Lornoxicam, Loxoprofen sodium hydrate, Mefenamic acid, Meloxicam, Nabumetone, Naproxen, Oxaprozin, Piroxicam, Planoprofen, Progourmet tacin maleate, Sulindac,
	Opioids		Buprenorphine, Fentanyl, Fentanyl citrate, Tramadol hydrochloride
	Others		Duloxetine hydrochloride, Purified sodium hyaluronate, Sodium hyaluronate cross-linked polymer/Sodium hyaluronate cross-linked polymer Vinyl sulfone cross-linked product, Vaccinia virus inoculated rabbit inflammation skin extract

bDMARD, biological disease-modifying antirheumatic drug; GC, glucocorticoid; csDMARD, conventional synthetic disease-modifying antirheumatic drug; IL6i, interleukin-6 inhibitor; MTX, methotrexate; NSAID, non-steroidal anti-inflammatory drug; RA, rheumatoid arthritis; T, selective T-cell costimulation modulator; TNFi, tumor necrosis factor α inhibitor; tsDMARD, targeted synthetic disease-modifying antirheumatic drug
