# Supplementary material for: Characteristics of medical costs and resource use in patients with rheumatoid arthritis treated with and without glucocorticoids
Source: PLoS One. 2025 Jul 30;20(7):e0329313. doi: 10.1371/journal.pone.0329313 (PMC12310026; doi:10.1371/journal.pone.0329313)
Supplement: S3 Table — (PDF) [file pone.0329313.s003.pdf]

S3 Table. Items included in drug costs for treatment of adverse events

Drug type		Ingredient name
Antibiotics	Antibacterial drugs	Aluminoparaaminosalicylic acid calcium hydrate, Amikacin sulfate, Amoxicillin hydrate, Amoxicillin hydrate/potassium clavulanate, Ampicillin hydrate, Ampicillin hydrate/Cloxacillin sodium hydrate, Ampicillin sodium, Ampicillin sodium/cloxacillin sodium hydrate, Ampicillin sodium/sulbactam sodium, Arbekacin sulfate, Azithromycin hydrate, Aztreonam, Bacampicillin hydrochloride, Bacitracin/fradiomycin sulfate, Bedaquiline fumarate, Benzyl penicillin potassium, Benzylpenicillin benzathine hydrate, Betamethasone sodium phosphate/fradiomycin sulfate, Betamethasone valerate/fradiomycin sulfate, Betamethasone valerate/gentamicin sulfate, Bezlotoxumab, Calcium para-aminosalicylate hydrate, Cefaclor, Cefazolin sodium, Cefazolin sodium hydrate, Cefcapene pivoxil hydrochloride hydrate, Cefdinir, Cefditoren pivoxil, Cefepime hydrochloride hydrate, Cefixime, Cefloxazine hydrate, Cefmenoxime hydrochloride, Cefmetazole sodium, Cefminox sodium hydrate, Cefoperazone sodium/sulbactam sodium, Cefotaxime sodium, Cefotiam hydrochloride, Cefozopran hydrochloride, Cefpirom sulfate, Cefpodoxime Proxetil, Ceftazidime hydrate, Cefteram Pivoxil, Ceftibutene hydrate, Ceftizoxime sodium, Ceftriaxone sodium hydrate, Ceftrozan sulfate/tazobactam sodium, Cefuroxime axetil, Cephalexin, Cephalotin sodium, Chloramphenicol, Chloramphenicol sodium succinate, Chloramphenicol/colistin sodium methanesulfonate, Chloramphenicol/fradiomycin combination, Ciprofloxacin, Ciprofloxacin hydrochloride, Citafloxacin hydrate, Clarithromycin, Clindamycin hydrochloride, Clindamycin phosphate, Clindamycin phosphate hydrate/benzoyl peroxide, Clofazimine, Colistin sodium methanesulfonate, Cycloserine, Daptomycin, Delamanide, Demethylchlortetracycline hydrochloride, Dibekacin sulfate, Diphenyl sulfone, Doripenem hydrate, Doxycycline hydrochloride hydrate, Enbiomycin sulfate, Epidihydrocholesterin/tetracycline hydrochloride, erythromycin, Erythromycin ethyl succinate, Erythromycin lactobionate, Erythromycin lactobionate/colistin sodium methanesulfonate, Erythromycin stearate, Ethambutol hydrochloride, Ethionamide, Faropenem sodium hydrate, Fidaxomycin, Flomoxef sodium, Fluocinolone acetonide/fradiomycin sulfate, Fosfomycin calcium hydrate, Fosfomycin sodium, Fradiomycin sulfate, Fradiomycin sulfate/methylprednisolone, Fradiomycin sulfate/trypsin, Galenoxacin mesylate hydrate, Gentamicin sulfate, Hexamine, Hydrocortisone acetate/fradiomycin combination, Hydrocortisone/fradiomycin combination, Imipenem hydrate/cilastatin sodium, Isepamicin sulfate, Isoniazid, Isoniazid sodium methanesulfonate hydrate, Josamycin, Josamycin propionate, Kanamycin monosulfate, Kanamycin sulfate, Lansoprazole/amoxicillin/clarithromycin, Lansoprazole/Amoxicillin/Metronidazole, Latamoxef sodium, Levofloxacin hydrate, Lincomycin hydrochloride hydrate, Linezolid, Lomefloxacin hydrochloride, Meropenem hydrate, Metronidazole, Minocycline hydrochloride, Moxifloxacin hydrochloride, Mupirocin calcium hydrate, Nadifloxacin, Norfloxacin, Ofloxacin, Oxytetracycline hydrochloride, Oxytetracycline hydrochloride/hydrocortisone, Oxytetracycline hydrochloride/Polymyxin B sulfate, Panipenem/Betamipron, Pazufloxacin mesylate, Pipemidic acid hydrate, Piperacillin sodium, Polymyxin B sulfate, Purrifloxacin, Pyrazinamide, Quinupristin/Dalhopristin, Rabeprazole sodium/amoxicillin hydrate/clarithromycin, Rabeprazole sodium/amoxicillin hydrate/metronidazole, Rifabutin, Rifampicin, Roxithromycin, Sodium fusidate, Spectinomycin hydrochloride hydrate, Spiramycin, Spiramycin acetate, Streptomycin sulfate, Sulfamethoxazole/trimethoprim, Sultamicillin tosylate hydrate, [illegible]
	Antifungal drugs	Amorolfine hydrochloride, Amphotericin B, Atobacon, Bifonazole, Butenafine hydrochloride, Caspofungin acetate, Clotrimazole, Efinaconazole, Fluconazole, Flucytosine, Fosfluconazole, Isoconazole nitrate, Itraconazole, Ketoconazole, Lilanaftate, Luliconazole, Micafungin sodium, Miconazole, Miconazole nitrate, Neticonazole hydrochloride, Oxyconazole nitrate, Pentamidine isethionate, Phoslabuconazole L-lysine ethanol adduct, Ranoconazole, Sulconazole nitrate, Terbinafine
	Antiviral drugs	Abacavir Sulfate, Acyclovir, Adefovir Pivoxil, Amantadine hydrochloride, Amena Building, Asuna Previr, Atazanavir sulfate, Daclatasvir hydrochloride, Daclatasvir Hydrochloride/Asunaprevir/Veclavvir Hydrochloride, Darunavir ethanol adduct, Darunavir ethanol adduct/cobicistat, Darunavir ethanol adduct/cobicistat/emtricitabine/tenofovir alafenamide fumarate, Dolutegravir sodium, Dolutegravir Sodium / Abacavir Sulfate / Lamivudine, Dolutegravir sodium/rilpivirine hydrochloride, Efavirenz, Elvitegravir/cobicistat/emtricitabine/tenofovir alafenamide fumarate, Elvitegravir/cobicistat/emtricitabine/tenofovir disoproxil fumarate, Emtricitabine, Emtricitabine/Tenofovir Alafenamide Fumarate, Emtricitabine/tenofovir disoproxil fumarate, Entecavir hydrate, Etrabilin, Famciclovir, Fosamprenavir calcium hydrate, Foscarnet sodium hydrate, Ganciclovir, Glazoprevir hydrate, Grecaprevir hydrate/pibrentasvir, Hervasville, Inosine Planovex, Lamivudine, Lamivudine/abacavir sulfate, Laninamivir octanoate hydrate, Lettermobil, Lopinavir/ritonavir, Maravirok, Nelfinavir mesylate, Nevirapine, Ombitasvir hydrate/paritaprevir hydrate/ritonavir, Oseltamivir phosphate, Palivizumab, Peramivir hydrate, Raltegravir potassium, Regipasvir Acetone adduct/Sofosbuvir, Ribavirin, Rilpivirine hydrochloride, Rilpivirine hydrochloride/emtricitabine/tenofovir alafenamide fumarate, Rilpivirine hydrochloride/emtricitabine/tenofovir disoproxil fumarate, Ritonavir, Sofosbuville, Sohosubu Building/Belpatas Building, Tenofovir alafenamide fumarate, Tenofovir disoproxil fumarate, Valacyclovir hydrochloride, Valacyclovir hydrochloride hydrate, Valganciclovir hydrochloride, Valoxavir marboxil, Victegravir sodium/emtricitabine/tenofovir
	Antiparasitic drugs	Albendazole, Artemether/Lumefantrine, Atovacon/Proguanil hydrochloride, Diethylcarbamazine citrate, Ivermectin, Mebendazole, Mefloquine hydrochloride, Metronidazole, Paromomycin sulfate, Praziquantel, Primaquine phosphate, Pyranterpamoate, Quinine ethyl carbonate, Quinine hydrochloride hydrate, Quinine sulfate hydrate, Tinidazole
Anti-osteoporotic drugs		Alendronate sodium hydrate, Alfa calcidol, Bazedoxifene acetate, Calcitriol, Calcium hydrogen phosphate hydrate, Denosumab, Disodium etidronate, Elcatonin, Eldecalcitol, Estradiol, Estradiol/Levonorgestrel, Estriol, Ibandronate sodium hydrate, Ipriflavone, L-calcium aspartate hydrate, Menatetrenone, Minodronic acid hydrate, Raloxifene hydrochloride, Risedronate sodium hydrate, Romosozumab, Teriparatide, Teriparatide acetate, Testosterone enanthate/estradiol valerate,
