# Supplementary material for: Characteristics of medical costs and resource use in patients with rheumatoid arthritis treated with and without glucocorticoids
Source: PLoS One. 2025 Jul 30;20(7):e0329313. doi: 10.1371/journal.pone.0329313 (PMC12310026; doi:10.1371/journal.pone.0329313)
Supplement: S4 Table — (PDF) [file pone.0329313.s004.pdf]

S4 Table. Items included in treatment costs

Treatment	Example of detailed item name
Outpatient	Outpatient fee
Hospitalization	Addition of basic hospital charges, Basic admission fee, Basic fee for short-term stay surgery, Specific hospitalization fee
Medical management	Medical management fee
Home health care	Home care and guidance fees, Home care guidance management fee
Examination	Biopsy fee, Diagnostic puncture/sample collection fee, Examination fee, specimen examination fee
Imaging	Computer tomography diagnostic fee, Image diagnosis fee, Nuclear medicine diagnostic fee, X-ray diagnostic fee
Medication	Dispensing fee, Dispensing technology basic fee, Prescription fee
Injection	Injection fee
Rehabilitation	Rehabilitation fee
Psychiatric specialty therapy	Psychiatric specialty therapy fee
Procedure	Addition of medical treatment equipment, Treatment fee
Surgery	Blood transfusion fee, Surgery fee, Addition of surgical medical equipment
Anesthesia	Anesthesia fee, Nerve block fee
Radiation therapy	Radiation treatment management/execution fee
Pathological diagnosis	Pathological diagnosis/judgment fee, Pathological specimen preparation fee
Others	Others
