# Supplementary material for: Characteristics of medical costs and resource use in patients with rheumatoid arthritis treated with and without glucocorticoids
Source: PLoS One. 2025 Jul 30;20(7):e0329313. doi: 10.1371/journal.pone.0329313 (PMC12310026; doi:10.1371/journal.pone.0329313)
Supplement: S5 Table — (PDF) [file pone.0329313.s005.pdf]

S5 Table. Items included in material costs

Example of detailed item name
Examination/diagnostic imaging materials
Injection/anesthesia materials
Medication specific-insurance medical materials
Continuous injection/drainage/exhaust conduit
Urinary/biliary materials
Blood purification method materials
Skeletal materials
Ophthalmology/otorhinolaryngology materials
Skin/tissue materials
Cardiovascular materials
Gastric/esophageal materials
Home healthcare-specific insurance medical materials
Imaging diagnosis-specific insurance medical materials
Other materials
