# Supplementary material for: Characteristics of medical costs and resource use in patients with rheumatoid arthritis treated with and without glucocorticoids
Source: PLoS One. 2025 Jul 30;20(7):e0329313. doi: 10.1371/journal.pone.0329313 (PMC12310026; doi:10.1371/journal.pone.0329313)
Supplement: S6 Table — (PDF) [file pone.0329313.s006.pdf]

S6 Table. The number and percentage of eligible patients and annual medical costs per patientby the disease name included in the Charlson comorbidity index and Charlson comorbidity index score

Patient characteristic	GC (N = 1,670)				Non GC (N = 1,487)			
	Number of patients		Annual medical costs		Number of patients		Annual medical costs	
	n	%	mean (SD) (USD/year)	median (IQR) (USD/year)	n	%	mean (SD) (USD/year)	median (IQR) (USD/year)
**Disease included in Charlson comorbidity index**								
Myocardial infarction	24	1.44	5,707 (5,780)	3,166 (2,253 - 5,763)	20	1.34	5,447 (8,404)	2,423 (2,016 - 3,148)
Congestive heart failure	140	8.38	7,875 (8,683)	3,987 (2,702 - 10,487)	103	6.93	7,138 (9,905)	2,894 (2,095 - 10,726)
Peripheral vascular disease	120	7.19	7,491 (11,161)	3,514 (2,321 - 9,515)	102	6.86	6,241 (8,376)	2,915 (1,908 - 5,653)
Cerebrovascular disease	99	5.93	7,508 (10,532)	3,538 (2,351 - 8,379)	74	4.98	5,792 (6,878)	2,948 (2,127 - 5,083)
Dementia	1	0.06	25,038 (NA)	25,038 (25,038 - 25,038)	0	0.00	NA	NA
Chronic lung disease	335	20.06	6,816 (8,662)	3,325 (2,171 - 8,205)	201	13.52	4,535 (6,862)	2,398 (1,677 - 3,598)
Collagen disease/connective tissue disease	1670	100.00	5,295 (6,797)	2,723 (1,820 - 5,679)	1487	100.00	3,817 (5,324)	2,100 (1,395 - 3,271)
Digestive ulcer	1	0.06	4,436 (NA)	4,436 (4,436 - 4,436)	6	0.40	9,802 (19,674)	1,851 (1,605 - 2,492)
Mild liver disease	713	42.69	6,118 (7,404)	3,091 (2,044 - 7,514)	651	43.78	4,156 (5,877)	2,317 (1,618 - 3,664)
Diabetes (no major complications, excluding dietary therapy only)	137	8.20	7,588 (10,606)	3,646 (2,419 - 9,089)	111	7.46	5,048 (6,055)	2,708 (1,932 - 5,242)
Hemiplegia	4	0.24	6,122 (5,084)	3,721 (3,516 - 6,327)	2	0.13	3,853 (840)	3,853 (3,556 - 4,150)
Moderate to severe renal dysfunction	226	13.53	7,474 (9,740)	3,339 (2,287 - 8,739)	157	10.56	5,466 (8,428)	2,656 (1,805 - 4,291)
Diabetes (any of the major complications, history of hospitalization for DKA or diabetic co	9	0.54	8,934 (7,692)	6,988 (3,026 - 10,880)	5	0.34	5,069 (1,600)	5,453 (3,449 - 6,351)
Solid cancer	284	17.01	6,564 (7,327)	3,549 (2,303 - 8,153)	234	15.74	4,660 (5,969)	2,575 (1,806 - 4,223)
Leukemia	15	0.90	7,776 (8,126)	3,166 (2,304 - 11,662)	9	0.61	5,146 (5,143)	2,828 (2,332 - 4,188)
Lymphoma	89	5.33	8,299 (7,743)	4,076 (2,510 - 13,401)	52	3.50	7,314 (11,015)	2,862 (1,979 - 5,776)
Moderate to severe liver dysfunction	154	9.22	5,479 (6,153)	2,845 (2,045 - 5,795)	128	8.61	4,085 (5,686)	2,083 (1,319 - 3,531)
Metastatic solid cancer	5	0.30	5,190 (6,357)	2,817 (2,321 - 2,840)	8	0.54	5,439 (3,114)	5,438 (3,643 - 6,887)
AIDS	0	0.00	NA	NA	0	0.00	NA	NA
Charlson comorbidity index score								
1	514	30.78	4,064 (4,815)	2,195 (1,445 - 4,094)	471	31.67	3,203 (4,315)	1,685 (1,113 - 2,586)
2	397	23.77	4,966 (5,553)	2,661 (1,764 - 5,442)	394	26.50	3,554 (4,781)	1,999 (1,400 - 3,148)
3	210	12.57	5,766 (9,379)	2,815 (1,988 - 4,994)	180	12.10	3,786 (4,504)	2,330 (1,694 - 3,199)
4	228	13.65	5,386 (5,710)	3,072 (2,044 - 5,969)	225	15.13	3,552 (4,539)	2,182 (1,501 - 3,128)
5	116	6.95	5,407 (6,843)	3,011 (2,119 - 6,565)	85	5.72	4,945 (5,976)	2,624 (1,538 - 4,333)
6	78	4.67	7,243 (9,638)	3,234 (2,644 - 8,686)	57	3.83	5,797 (9,340)	2,859 (2,008 - 3,935)
7	46	2.75	8,390 (11,141)	3,676 (2,546 - 8,157)	30	2.02	5,228 (8,802)	2,741 (1,998 - 4,247)
8	30	1.80	9,189 (9,800)	4,475 (2,755 - 15,059)	21	1.41	5,677 (5,477)	3,011 (2,422 - 5,453)
9 or more	51	3.05	9,602 (8,135)	7,060 (2,647 - 15,874)	24	1.61	10,816 (12,413)	6,344 (2,946 - 14,421)
